# The Influence of Format Familiarity on the Word Segmentation Effect in Tibetan Reading

**DOI:** 10.3390/bs16071119

**Published:** 2026-07-03

**Authors:** Hongyu Liang, Chenxu Zhang, Zijian Xie, Lei Gao, Xiaolei Gao

**Affiliations:** Plateau Brain Science Research Center, Xizang University, Lhasa 850000, China; 18228247975@163.com (H.L.); zcx129511@163.com (C.Z.); xiezijian08@163.com (Z.X.); gaolei1983good@163.com (L.G.)

**Keywords:** visual cues, format familiarity, Tibetan reading, eye movements

## Abstract

To examine the effect of format familiarity on word segmentation in Tibetan reading, this study manipulated both format familiarity and visual cues. An EyeLink 1000Plus eye tracker was used to record the eye movement characteristics of 75 Tibetan college students during Tibetan reading tasks. The results reveal that, under conditions of low format familiarity, inter-word spaces significantly facilitated Tibetan reading; however, this facilitative effect disappeared as format familiarity increased. These findings suggest that, in Tibetan reading, there is a trade-off between format familiarity and the facilitating effect of inter-word spaces.

## 1. Introduction

### 1.1. Research Rationale

Reading is a highly automated human cognitive activity. One core component of reading is word segmentation—the process of identifying word boundaries and extracting independent word units from continuous text by relying on either explicit visual cues or implicit linguistic information (L. Gao et al., 2024; L. Gao et al., 2022; X. L. Gao et al., 2024; X. L. Gao et al., 2022). The efficiency of word segmentation directly determines reading fluency and exhibits cross-linguistic universality. In languages with spaces, such as English, inter-word spaces serve as explicit word-segmentation cues, reducing the number of fixations and shortening total fixation time (Pollatsek & Rayner, 1982; Rayner, 2009; Rayner et al., 1998; Sheridan et al., 2013). A large body of cross-linguistic research has confirmed that inter-word spaces play a fundamental role in regulating eye movements and lexical processing for alphabetic readers (Drieghe et al., 2017; Veldre et al., 2017). In languages without spaces, such as Japanese and Hebrew, readers rely on implicit cues, such as character shapes and prosodic patterns, to perform word segmentation. If spaces are artificially inserted into their texts, processing efficiency can be significantly enhanced (X. Bai et al., 2011; Koriat & Greenberg, 1994; Sainio et al., 2007). To further verify this pattern across regions and script types, existing eye-tracking studies on three typical unspaced alphabetic languages—Japanese, Korean and Thai—have all reached consistent conclusions about the facilitative function of inter-word spaces (Chantavarin & Leung, 2025; Cho & Gordon, 2014; Kajii et al., 2001). Chinese is also a typical language without spaces. Early studies found that Chinese readers depend on implicit cues, including word frequency, syntax, and statistical learning, to accomplish lexical segmentation (Sun et al., 1985; Yan et al., 2012). With advances in experimental techniques, researchers have begun to examine the role of explicit segmentation cues through artificial interventions, such as inserting inter-word spaces. Some studies have shown that adding inter-word spaces does not significantly shorten the total reading time or increase reading speed among adult native Chinese speakers; in some cases, it even leads to slightly slower reading due to interference caused by an unfamiliar format (Y. Bai et al., 2008; Ma et al., 2014; Rayner et al., 2013). However, the intervention proves highly beneficial for second-language learners and children (S. Li et al., 2022; Pan et al., 2021; Shen et al., 2012; Zang et al., 2013; Zhou et al., 2018).

A variety of factors influence the spacing effect. For instance, in the reading of highly difficult texts, the facilitating role of spaces may be diminished (Rayner et al., 2006). Another key moderator is format familiarity, which refers to readers’ familiarity with a given text layout or reading direction. A typical example is reversed reading (from right to left), which differs from the conventional left-to-right layout. Readers who are accustomed to standard reading directions produce shorter saccades (rapid eye movements between fixations) and more regressions (backward eye movements for rechecking content) when facing reversed text. This phenomenon fully reflects readers’ dependence on familiar reading formats (Pollatsek et al., 1981). Building on this line of work, Y. Bai et al. (2008) and Y. Wang et al. (2022) investigated reading direction and word spacing in Chinese reading. The results showed that, under conventional reading conditions, reading from left to right, the addition of inter-word spaces did not significantly enhance or hinder reading performance. From these findings, the researchers proposed the trade-off hypothesis. This hypothesis states that two forces interact dynamically during reading: the facilitating effect of explicit segmentation cues and the interfering effect of unfamiliar text formats. When readers are highly familiar with text format, they can use automated implicit segmentation strategies to finish word recognition and sentence comprehension. In unfamiliar reversed reading conditions, however, readers show longer fixations and more regressions from the early stage of text processing (Y. Bai et al., 2008; Cao et al., 2023; X. Li et al., 2010).

Tibetan is a phonetic script that is written from left to right and uses the syllable separator “·” (tsek) to mark syllable boundaries. While the syllable separator aids in syllable segmentation, it does not provide reliable information about word boundaries, which leads to considerable ambiguity in word segmentation during Tibetan reading (Tournadre, 2014). Consequently, when reading Tibetan, readers must rely on lexical, syntactic, and contextual cues to segment words (X. Bai et al., 2017; D. H. Wang et al., 2023, 2024). Some researchers have examined the impact of visual segmentation cues on Tibetan reading. D. H. Wang et al. (2023) found that the native syllable separator in Tibetan can effectively assist readers in syllable segmentation; although replacing it with spaces did not yield stronger advantages than the original separator at the level of syllable recognition, it did further enhance overall sentence-reading efficiency, for instance, by reducing fixation duration and increasing reading speed, possibly due to its ability to alleviate the visual crowding effect. Subsequently, D. H. Wang et al. (2024) further investigated the role of inter-word spaces in Tibetan reading and found that artificially inserting spaces between words not only promotes reading efficiency and lexical recognition but also helps readers make more accurate saccade target selections. These studies provide additional evidence for the mechanisms underlying the effects of word segmentation in phonetic scripts (X. L. Gao et al., 2020; S. Li et al., 2022; X. Li et al., 2022). Collectively, these prior findings have deepened our understanding of how visual spacing cues operate in Tibetan and other phonetic languages, forming a solid theoretical foundation for the present research.

Despite the above progress, existing Tibetan reading studies have only explored the general effects of various spacing settings, without considering how readers’ familiarity with text formats modulates the function of word segmentation cues. The trade-off hypothesis proposed by Y. Bai et al. (2008) posits that the facilitative effect of explicit segmentation cues and the interfering effect of unfamiliar text formats interact dynamically during reading. This hypothesis has been fully validated in Chinese reading research (Y. Wang et al., 2022), yet it has never been systematically tested in phonetic writing systems like Tibetan. To address this research gap, the present study conducts relevant verification with Tibetan participants. Meanwhile, Tibetan, as a phonetic script, possesses unique characteristics that set it apart from other phonetic scripts. In addition to its phonetic features, Tibetan belongs to the Sino-Tibetan language family and shares close linguistic ties with Chinese (X. Bai et al., 2017; Yan et al., 2013). Thus, it remains to be seen whether the trade-off hypothesis proposed by Y. Bai et al. (2008) based on Chinese reading will be revalidated given Tibetan’s Chinese-like features, or whether Tibetan’s status as a phonetic script with inherent phonetic characteristics will give rise to certain unique patterns. These questions warrant further empirical investigation and theoretical exploration.

Clarifying these issues can not only fill the research gap regarding the cross-script generalizability of the trade-off hypothesis, but also advance Tibetan reading research, and further connect findings of script-specific word segmentation mechanisms with universal eye-movement control models—such as the E-Z Reader model, a widely adopted computational model for simulating human eye movement control during reading.

### 1.2. Theoretical and Practical Implications

Across different cultures and language families, readers adopt diversified reading strategies to adapt to their native writing systems. Visual cues and format familiarity are two universal factors shaping reading behaviors. Existing studies have discussed spacing effects in alphabetic, logographic and other scripts, but targeted empirical evidence from Tibetan remains insufficient.

The present research carries dual theoretical and practical value. Theoretically, verifying the trade-off hypothesis on Tibetan can enrich cross-linguistic reading theories and link script-specific experimental results with classic eye-movement simulation frameworks. Practically, the results can provide empirical references for layout design of Tibetan textbooks, reading materials and daily Tibetan literacy teaching.

### 1.3. Research Purpose and Experimental Design

Drawing on the experimental designs of Y. Wang et al. (2022) and Chen et al. (2022), this study recruited Tibetan college students as participants and adopted eye-tracking technology to manipulate two core variables: visual segmentation cues and format familiarity.

1. Unfamiliar reading context: We created reversed right-to-left Tibetan text to reduce format familiarity, and set four visual spacing conditions: unspaced, inter-word spaced, inter-character spaced, non-word spaced, to observe spacing facilitation under low familiarity.
2. Familiarity training procedure: Participants received 10 consecutive days of reversed reading training (30 min per day). Post-training reading tests under identical spacing treatments represented the familiar format condition.

By comparing eye movement indicators before and after training, we aimed to examine whether the facilitative effect of inter-word spaces diminishes or disappears as format familiarity increases, which generates two core research questions.

### 1.4. Research Hypotheses

To answer the two core research questions, two testable hypotheses are listed in bullet points for intuitive reading:

**Hypothesis 1** **(H1).** *Under the unfamiliar reversed reading format, inter-word spaces will significantly improve Tibetan reading efficiency compared with the unspaced control condition*.

**Hypothesis 2** **(H2).** *After systematic reversed reading training that elevates format familiarity, the facilitative effect of inter-word spaces will significantly weaken or even vanish*.

## 2. Materials and Methods

### 2.1. Experimental Design

The experiment employed a 2 (format familiarity: unfamiliar format, familiar format) × 4 (visual cues: unspaced, inter-word spaced, inter-character spaced, non-word spaced) mixed-factor experimental design. Format familiarity was a between-subjects variable: from the reader’s perspective, if the reader had not undergone reverse reading training, reverse reading would be performed on materials that were, for the reader, unfamiliar format; conversely, if the reader had undergone reverse reading training, the materials would be considered familiar format. Visual cues were a within-subjects variable: in the unspaced condition, there were no spaces within the sentence; in the inter-word spaced condition, spaces were inserted between words; in the inter-character spaced condition, spaces were inserted between adjacent characters; and in the non-word spaced condition, random spaces were inserted throughout the sentence, thereby transforming adjacent words into non-word entities.

### 2.2. Participants

Following Cohen (1988) and Faul et al. (2009) regarding power analysis for mixed designs, we set the statistical power to 0.8, the medium effect size (*f* = 0.25), and the within-variable correlation to 0.5. G*Power 3.1.9.7 calculated that at least 36 participants (18 per group) were required. Considering potential invalid participants, we recruited 81 Tibetan college students from Xizang University. They were divided into two groups: the Untrained Group and the Training Group. The Untrained Group consisted of 41 participants (Mean age = 19.90 ± 0.97 years, 35 females, 6 males). The Training Group consisted of 40 participants; after 10 days of training, only 34 participants completed all training and subsequent eye-tracking tests (Mean age = 19.85 ± 1.08 years, 30 females, 4 males). Ultimately, 75 Tibetan college students participated in the experiment (Mean age = 19.88 ± 1.01 years, 65 females, 10 males). There was no significant difference in college entrance exam Tibetan scores between the Untrained Group (Mean = 130.80 ± 7.69) and the Training Group (Mean = 132.97 ± 6.17), *t* = 1.33, *p* = 0.189, indicating no difference in Tibetan reading ability. All participants were native Tibetan speakers, had normal cognitive development, normal or corrected-to-normal vision, no physical or mental illnesses, no reading disorders, and were right-handed. The experiment was approved by the Ethics Committee of Xizang University (IRB NO: XZDXLL2022016). All participants signed informed consent forms before the experiment, participated voluntarily, and received compensation after the experiment.

### 2.3. Experimental Materials

Referring to the previous literature (D. H. Wang et al., 2023), we adapted and refined 120 Tibetan declarative sentences based on university-level Tibetan textbooks and extracurricular reading materials of comparable difficulty. All sentences were carefully designed to avoid syntactic or semantic ambiguity, with sentence lengths controlled within a range of 25 to 30 standard character spaces. After the sentences were finalized, 16 Tibetan college students rated their difficulty and fluency using a five-point scale. The rating results are as follows: Difficulty: *M* = 1.80, *SD* = 0.92, 1 = very easy; Fluency: *M* = 2.10, *SD* = 1.15, 1 = very fluent. In addition, we performed word segmentation on the above sentences according to the criteria outlined in the “Modern Tibetan Frequency Dictionary” (Lu & Gao, 2007). Furthermore, the same 16 Tibetan college students evaluated the consistency of word segmentation. The results showed that the agreement rate reached 86.03%. These evaluation findings indicate that all 120 candidate Tibetan sentences meet the experimental requirements. We screened and retained 88 sentences with homogeneous length distribution to control potential length-related confounding effects (mean sentence length: *M* = 27.03, *SD* = 2.09). Of these, 80 sentences were designated as formal experimental sentences, while the remaining 8 served as practice sentences.

We created four visual cue conditions by inserting spaces into the selected sentences: unspaced, inter-word spaced, inter-character spaced, and non-word spaced. For the inter-character spaced condition, we used half-width spaces generated by the Tibetan input method. The visual width of these spaces is equivalent to that of the standard Tibetan syllable separator Tsheg (·). Spaces in this condition were inserted between every two adjacent graphemes regardless of syllabic boundaries, which only altered visual density without providing valid linguistic segmentation cues. Once the experimental sentences were finalized, Tibetan students majoring in Tibetan language and literature modified their formatting, changing the format from left-to-right to right-to-left. None of the students involved in material preparation, rating, or format modification participated in the subsequent formal experiment.

In the formal experiment, the experimental sentences were presented under four conditions, as shown in Table 1. Each condition included 20 formal experimental sentences and 2 practice sentences. A Latin square design was used to determine the order in which the sentences were presented under each condition, thereby creating four blocks. Each participant read only the experimental sentences presented in one specific order, that is, one block. In addition, to ensure that participants carefully read and correctly understood the meaning of the sentences, a comprehension question followed every 30 sentences. Participants were required to answer “yes” or “no” to these questions; half of the answers were “yes” and the other half were “no”.

The reading practice materials are drawn from Chinese textbooks for primary and secondary schools as well as extracurricular readings of comparable difficulty, and have been appropriately adapted to form 50 Chinese passages covering various fields such as daily life, culture, science, and history. Two Tibetan graduate students majoring in Tibetan-Chinese translation from the School of Literature translated the above-mentioned 50 Chinese texts into Tibetan and conducted proofreading. Subsequently, the text direction was reversed, presented from right to left, to encourage readers to read from right to left. The average number of Tibetan characters in each translated Tibetan passage was *M* = 202.42 (*SD* = 13.99). Each Tibetan passage is followed by seven reading comprehension questions, including three detailed-comprehension questions, two inference questions, and two main-idea questions. Participants were required to select the best answer from among four options for each question.

### 2.4. Experimental Apparatus

This eye-tracking experiment was designed to explore the interactive effect between text format familiarity and inter-word visual segmentation cues in Tibetan reading. We selected the EyeLink 1000Plus binocular eye tracker produced by SR Research (Ottawa, ON, Canada) for data recording, as this device delivers high 1000 Hz sampling precision and standardized cognitive analysis algorithms that are universally adopted in reading eye-movement research, which guarantees stable and reliable capture of fixations, saccades and regressions.

- Eye tracker model: EyeLink 1000Plus (SR Research, Canada).
- Sampling rate: 1000 Hz.
- Saccade detection thresholds (Liao et al., 2022; D. H. Wang et al., 2023):
  (1) Saccade velocity threshold: 30°/s.
  (2) Saccade acceleration threshold: 8000°/s^2^.
  (3) Saccade amplitude threshold: 0.1°.
- Display monitor: 24.5-inch DELL monitor (Dell Technologies, Round Rock, TX, USA):
  (1) Refresh rate: 240 Hz.
  (2) Screen resolution: 1920 × 1080 pixels.
  (3) Fixed viewing distance: 65 cm from participants’ eyes to screen.

The unified monitor configuration (fixed screen size, refresh rate, resolution and viewing distance) standardized visual input across all participants. This strict consistent display setup eliminates visual-angle variation as confounding noise, ensuring that any differences in eye-movement indicators only stem from our manipulated experimental variables rather than inconsistent visual presentation.

For stimulus presentation, all Tibetan experimental texts adopted Microsoft Himalaya font with a 36-point size. This font type and size were deliberately chosen following established eye-tracking reading research on Tibetan scripts (S. Li et al., 2022): this setting generates a consistent 0.6° visual angle per Tibetan character, which avoids over-large visual load or overly tiny text that would induce extra oculomotor interference and matches the stimulus visual parameters widely used in comparable unspaced phonetic script reading experiments.

### 2.5. Experimental Procedure

The experiment involved two groups of participants: an untrained group and a trained group. Consequently, the experimental procedures differed for these two groups of participants.

Experimental procedure for the non-training group: (1) After entering the laboratory, participants first familiarized themselves with the experimental environment, then take their seats at the designated location. The experimenter briefly introduced the overall experimental procedure. (2) Instructions were displayed on the screen. Once participants have finished reading, the experimenter provided further details about the specific requirements of the experiment. (3) Eye-tracking calibration is performed. To ensure the accuracy of eye-tracking data recording, the experiment employed a three-point calibration method. (4) The formal experiment begins. During the experiment, participants were instructed to keep their heads as still as possible. At the start of each trial, the gaze point was presented on the right side of the screen’s center (aligned with the position of the first character in the sentence). The participant fixed their gaze on this point and pressed the page-turn key to proceed to the experiment. The experimental materials were presented sentence by sentence, with one sentence displayed per screen. After some sentences, there were comprehension questions that require the participant to respond by pressing a key. Before the formal experiment began, there was a practice session to help participants become familiar with the procedure and key-press tasks. During the experiment, if the participant needed to take a break, the experiment was paused. After the break, calibration was re-performed before resuming the reading of Tibetan sentences. Each participant typically takes about 30 to 45 min to complete this task.

**Figure 1:**
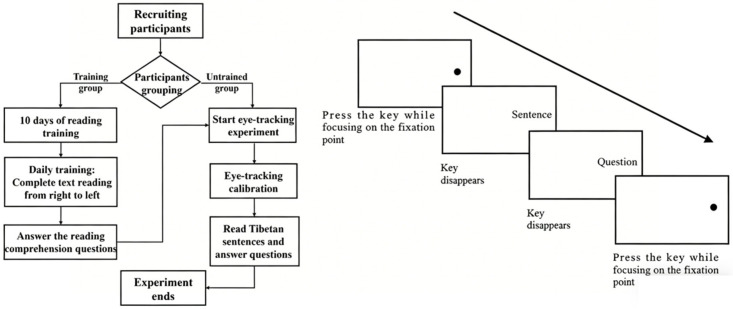
Experimental procedure flow chart.

The experimental procedure for the training group consists of two phases: a training phase and a testing phase. 1. Training Phase. The training lasts for 10 days, with the specific procedure as follows: (1) After entering the laboratory and familiarizing themselves with the environment, participants fill out basic information as required and take their seats. Each participant’ s seat was equipped with a test booklet containing passages to be read. The experimenter informed the participants that they should remain as quiet as possible throughout the training process. (2) Participants engage in reading practice. First, the experimenter reads out the instructions, explaining that the participants will be reading texts written and presented from right to left. Participants were instructed to read carefully, word by word and sentence by sentence, in a right-to-left order, and to fully comprehend the meaning of the text. Each passage was followed by seven reading comprehension questions. Participants must select the best answer based on the content of the passage and write the corresponding answer number in the designated space in the test booklet. After understanding the instructions, participants begin reading; upon completing each passage, they also complete the associated questions before moving on to the next one. (3) Each reading practice session lasts 30 min. Participants conduct one practice session per day, completing five passages in each session. The participants’ accuracy rate in answering the reading comprehension questions reached 87.6%, indicating that all subjects carefully read the Tibetan texts presented in reverse order. 2. Testing Phase. After completing the final day of training, each subject underwent a test. The experimental procedure during the testing phase is identical to that used in the non-training group. The overall experimental design and the flow of a single trial are illustrated in Figure 1.

### 2.6. Measures

Referring to the previous literature (Y. Bai et al., 2008; X. L. Gao et al., 2022; Rayner, 1998, 2009; D. H. Wang et al., 2023), the following eye-movement indicators were selected: (1) Reading Rate (RR, characters/s): Reflects overall reading fluency and automatization; lower reading rates indicate reading difficulties or a greater demand for cognitive resources. (2) Average Fixation Duration (AFD, ms): Reflects the overall efficiency of information extraction and processing; longer average fixation durations suggest that more time is required to process visual information or that there are comprehension difficulties. (3) Total Reading Time per Sentence (TRT, ms): Comprehensively reflects the overall cognitive load involved in sentence processing, including all stages of processing such as early visual encoding, lexical recognition, semantic integration, and regressions for correction. (4) Forward Saccade Probability (FSP, %): Reflects the smoothness of reading progression and expected processing efficiency; a higher forward saccade probability indicates that readers can smoothly integrate current information and make effective predictions about subsequent content, whereas a lower probability suggests local processing difficulties or reduced text predictability. (5) Regression Count (RSC, times): Reflects comprehension difficulties and monitoring/revision behaviors during reading; a higher number of regressions indicates that readers have encountered obstacles in processing current information and need to return to earlier content for reanalysis to overcome lexical, syntactic, or semantic processing challenges. (6) Total Number of Fixations (TNF, times): Reflects the overall cognitive effort and attention allocation during text processing; a greater number of fixations suggests that the text is more difficult or requires more processing resources.

## 3. Results

The average accuracy rate of the participants’ responses was 87.60%, indicating that the participants carefully read and comprehended the sentences. Referring to previous studies (Y. Bai et al., 2008; Liang et al., 2017; Liversedge et al., 2004; Rayner et al., 2003; D. H. Wang et al., 2023, 2024), we excluded data based on the following five criteria: (1) data with fixation durations shorter than 80 ms or longer than 1200 ms; (2) data in which participants accidentally pressed keys during the experiment, leading to experimental interruptions; (3) data lost unexpectedly due to head movements or other reasons; (4) data with fewer than 4 fixations; (5) data exceeding three standard deviations. The proportion of deleted data accounted for 2.98% of the total dataset.

Data analysis was conducted in the R environment (version 4.5.1), using the lme4 package to build linear mixed-effects models and generalized linear mixed-effects models (R Core Team, 2021). Prior to model fitting, we conducted data screening and transformation to satisfy the assumptions of mixed-effects models. All continuous eye-movement measures were log-transformed to address non-normality and meet the normality assumption of linear mixed-effects models. Specifically, reading rate (RR), average fixation duration (AFD), total sentence reading time (TRT), and total number of fixations (TNF) were analyzed using linear mixed-effects models (LMMs) as continuous variables; forward saccade probability (FSP), treated as a proportion variable, was analyzed using a binomial generalized linear mixed-effects model (GLMM) with a logit link function; and the number of regressions (RSC), treated as a count variable, was analyzed using a generalized linear mixed-effects model assuming either Poisson or negative binomial distributions. For model specification, format familiarity and three word-segmentation contrast terms were defined as fixed effects. We adopted a fully crossed random-effects structure, where both participants and experimental items were included as random intercepts to account for variability across individual readers and stimulus materials (Baayen et al., 2008). No random slopes were added due to model convergence considerations. The Markov-Chain Monte Carlo algorithm derives model parameters from the posterior distribution as estimates of significance, simultaneously accounting for variance both from participants and items (Baayen et al., 2008; Baayen & Milin, 2010). An absolute value of *t* greater than 1.96 at the 5% level indicates significance.

In this study, the visual cue variable comprises four levels. To avoid multiple-comparison bias and focus on the core research question (Maxwell et al., 2017), we did not include the visual cue as a four-level factor in the statistical model. Instead, drawing on the previous literature and guided by the theoretical framework, we integrated it into three contrast terms with clear theoretical implications, which were then coded (Y. Bai et al., 2008; Chen et al., 2022; Maxwell et al., 2017; Rosenthal et al., 2000). Consequently, the model examines the format familiarity, three word-segmentation contrasts, and the interaction between each word-segmentation contrast and text-familiarity. Specifically, Format-familiarity: unfamiliar format vs. familiar format; Word Segmentation 1: Inter-word spaced vs. Unspaced; Word Segmentation 2: Inter-character spaced vs. Unspaced; Word Segmentation 3: Non-word spaced vs. Unspaced. For each word-segmentation contrast, if a significant interaction is found between that contrast and format-familiarity, we conduct pairwise comparisons separately under both the familiar format and the unfamiliar format. If Word Segmentation 1 shows a significant interaction with format-familiarity (Interaction 1), we compare the inter-word spaced condition with the unspaced condition under the unfamiliar format condition (Comparison 1) and also compare the inter-word spaced condition with the unspaced condition under the familiar format condition (Comparison 4). If Word Segmentation 2 shows a significant interaction with format-familiarity (Interaction 2), we compare the s inter-character spaced condition with the unspaced condition under the unfamiliar format condition (Comparison 2) and also compare the inter-character spaced condition with the unspaced condition under the familiar format condition (Comparison 5). If Word Segmentation 3 shows a significant interaction with format-familiarity (Interaction 3), we compare the non-word spaced condition with the unspaced condition under the unfamiliar format condition (Comparison 3) and also compare the non-word spaced condition with the unspaced condition under the familiar format condition (Comparison 6).

Effect sizes were reported as equivalent correlation coefficient r. For outcomes derived from t statistics in linear mixed-effects models, the formula r = √(t^2 / (t^2 + df)) was adopted with an approximate degree of freedom of 73. For simple effects based on z tests, effect size was calculated as r = z / √N,  N = 75, total valid participants). According to conventional standards, r values of approximately 0.1 indicate small effects, 0.3 indicate medium effects, and 0.5 or above indicate large effects.

**Figure 2:**
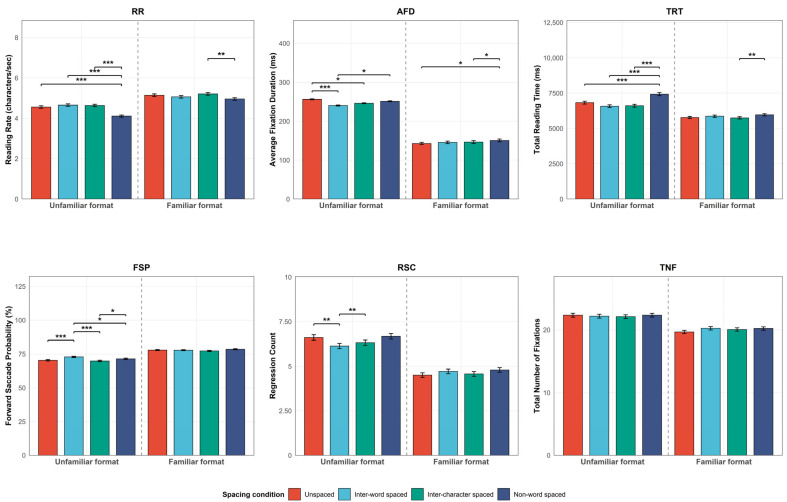
Bar charts of eye movement measures across conditions. Note. * *p* < 0.05, ** *p* < 0.01, *** *p* < 0.001.

The descriptive statistical results for each eye-movement index under different conditions are shown in Table 2; the statistical analysis results are presented in Table 3 and Figure 2.

The main effect of format familiarity was significant. As stated in the Introduction, our training paradigm was used to manipulate format familiarity. Compared to the unfamiliar format condition, the familiar format condition resulted in faster reading speed (*b* = 0.14, *SE* = 0.05, *t* = 2.67, *p* = 0.009, 95% CI = [0.04, 0.25], *r* = 0.30), shorter average fixation duration (*b* = −0.69, *SE* = 0.04, *t* = −17.00, *p* < 0.001, 95% CI = [−0.77, −0.61], *r* = 0.89), shorter total sentence reading time (*b* = −0.14, *SE* = 0.05, *t* = −2.58, *p* = 0.012, 95% CI = [−0.25, −0.03], *r* = 0.29), a higher probability of forward saccades (*b* = 0.42, *SE* = 0.12, *t* = 3.52, *p* < 0.001, 95% CI = [0.19, 0.66], *r* = 0.38), fewer regressions (*b* = −0.32, *SE* = 0.09, *t* = −3.54, *p* < 0.001, 95% CI = [−0.49, −0.14], *r* = 0.38), and fewer total fixations (*b* = −0.10, *SE* = 0.05, *t* = −2.07, *p* = 0.042, 95% CI = [−0.20, −0.01], *r* = 0.24). The main effect of word segmentation 1 was significant. Compared to the unspaced condition, the inter-word spaced condition resulted in faster reading speed (*b* = 0.03, *SE* = 0.01, *t* = 2.31, *p* = 0.021, 95% CI = [0.01, 0.06], *r* = 0.26), shorter average fixation duration (*b* = −0.07, *SE* = 0.01, *t* = −4.43, *p* < 0.001, 95% CI = [−0.10, −0.04], *r* = 0.46), shorter total sentence reading time (*b* = −0.03, *SE* = 0.01, *t* = −2.22, *p* = 0.026, 95% CI = [−0.05, −0.01], *r* = 0.25), a higher probability of forward saccades (*b* = 0.13, *SE* = 0.03, *t* = 4.50, *p* < 0.001, 95% CI = [0.07, 0.18], *r* = 0.47), and fewer regressions (*b* = −0.05, *SE* = 0.02, *t* = −2.11, *p* = 0.035, 95% CI = [−0.09, −0.01], *r* = 0.24). The main effect of word segmentation 2 was significant. Compared to the unspaced condition, the inter-character space condition resulted in shorter average fixation duration (*b* = −0.04, *SE* = 0.01, *t* = −2.82, *p* = 0.005, 95% CI = [−0.07, −0.01], *r* = 0.31). The main effect of word segmentation 3 was significant. Compared to the unspaced condition, the non-word space condition resulted in slower reading speed (*b* = −0.10, *SE* = 0.01, *t* = −7.81, *p* < 0.001, 95% CI = [−0.13, −0.08], *r* = 0.67); the total reading time for sentences was longer (*b* = 0.09, *SE* = 0.01, *t* = 6.84, *p* < 0.001, 95% CI = [0.06, 0.11], *r* = 0.62); and the probability of forward saccades was higher (*b* = 0.04, *SE* = 0.03, *t* = 2.42, *p* = 0.016, 95% CI = [0.01, 0.12], *r* = 0.27).

The interaction between word segmentation and format familiarity was significant, as evidenced by the following metrics: reading speed (*b* = −0.05, *SE* = 0.02, *t* = −2.38, *p* = 0.017, 95% CI = [−0.08, −0.01], *r* = 0.27); average fixation duration (*b* = 0.08, *SE* = 0.02, *t* = 3.44, *p* < 0.001, 95% CI = [0.04, 0.12], *r* = 0.37); total sentence reading time (*b* = 0.04, *SE* = 0.02, *t* = 2.12, *p* = 0.034, 95% CI = [0.01, 0.08], *r* = 0.24); probability of forward saccades (*b* = −0.13, *SE* = 0.04, *t* = −3.15, *p* = 0.002, 95% CI = [−0.21, −0.05], *r* = 0.35); and number of regressions (*b* = 0.08, *SE* = 0.03, *t* = 2.39, *p* = 0.017, 95% CI = [0.01, 0.15], *r* = 0.27). Simple effects analysis revealed that under unfamiliar format conditions, compared to the unspaced condition, the average fixation duration was longer in the inter-word spaced condition (comparison 1: *b* = 0.07, *SE* = 0.01, *z* = 4.43, *p* < 0.001, 95% CI = [0.04, 0.10], *r* = 0.48), the probability of forward saccades was higher (comparison 1: *OR* = 1.13, *SE* = 0.03, *z* = 5.05, *p* < 0.001, 95% CI = [1.07, 1.19], *r* = 0.55), and the number of regressions was lower (comparison 1: *OR* = 0.95, *SE* = 0.01, *z* = −3.70, *p* = 0.001, 95% CI = [0.93, 0.98], *r* = −0.40). However, in familiar format conditions, the differences between the inter-word spaced and unspaced conditions on these metrics were not significant. The interaction between word segmentation 3 and format familiarity was also significant, as reflected in the following metrics: reading speed (*b* = 0.06, *SE* = 0.02, *t* = 3.38, *p* < 0.001, 95% CI = [0.03, 0.10], *r* = 0.37); average fixation duration (*b* = 0.08, *SE* = 0.02, *t* = 3.18, *p* = 0.002, 95% CI = [0.03, 0.12], *r* = 0.35); and total sentence reading time (*b* = −0.05, *SE* = 0.02, *t* = −2.79, *p* = 0.005, 95% CI = [−0.09, −0.02], *r* = 0.31). Simple effects analysis showed that under unfamiliar format conditions, compared to the unspaced condition, the reading speed was slower in the non-word spaced condition (comparison 3: *b* = 0.10, *SE* = 0.01, *z* = 7.81, *p* < 0.001, 95% CI = [0.07, 0.13], *r* = 0.85), and the total sentence reading time was longer (comparison 3: *b* = −0.09, *SE* = 0.01, *z* = −6.84, *p* < 0.001, 95% CI = [−0.11, −0.06], *r* = −0.74). In contrast, under familiar format conditions, the average fixation duration was longer in the non-word spaced condition (comparison 6: *b* = −0.05, *SE* = 0.02, *z* = −2.90, *p* = 0.019, 95% CI = [−0.09, −0.02], *r* = −0.33).

The above results indicate that, under unfamiliar format conditions, inter-word spaces can effectively facilitate Tibetan reading, thereby supporting H1. Under familiar format conditions, however, the facilitating effect of inter-word spaces disappears, supporting H2. In sum, in Tibetan reading, there is a trade-off between the format familiarity and the facilitating effect of inter-word spaces.

## 4. Discussion

This study, conducted with Tibetan college students as participants and using eye-tracking technology, primarily explores the following two questions: (1) Under conditions where the text format is unfamiliar, does the presence of inter-word spaces facilitate Tibetan reading? (2) As format familiarity increases, will the facilitating effect of inter-word spaces diminish or even disappear altogether? The experimental results indicate that, under conditions of an unfamiliar format, word spacing significantly facilitates Tibetan reading; however, once format familiarity improves, this facilitating effect disappears.

### 4.1. Verification and Analysis of Hypothesis H1

The findings of this study first support H1. Under conditions of unfamiliar format, inter-word spaces significantly increased reading speed, reduced average fixation duration and total sentence reading time, while also boosting the probability of forward saccades and decreasing the number of regressions. This core finding is highly consistent with existing research results and represents an extension across different language systems. Specifically, the current results show a high degree of consistency with relevant findings from Chinese reading studies. For example, Y. Wang et al. (2022) observed the facilitating effect of inter-word spaced on Chinese reading under low-familiarity text conditions, while Chen et al. (2022) further concluded that “readers rely more heavily on explicit segmentation cues under low-familiarity conditions.” This study yielded an entirely consistent pattern of results in the Tibetan reading context, thereby confirming the cross-linguistic applicability of this cognitive principle. At the same time, this study provides empirical support from the Tibetan language for existing theoretical hypotheses. Y. Bai et al. (2008) previously proposed the hypothesis that “the facilitative and interfering effects produced by the format familiarity and the presence or absence of word boundary information may involve a trade-off.” This study verified that hypothesis using Tibetan reading data, demonstrating that this trade-off mechanism is universal across different language systems. Moreover, D. H. Wang et al. (2023) found that syllable boundaries play a moderating role in Tibetan reading, and D. H. Wang et al. (2024) further confirmed the general facilitating effect of inter-word spaces on Tibetan reading. Building on these findings, this study clarifies further that this facilitating effect becomes more pronounced when the format familiarity is lower, thus refining our understanding of the conditions under which visual word-segmentation cues operate in Tibetan reading (L. Li et al., 2025).

From a theoretical perspective, the significant facilitating effect of inter-word spaces under low-familiarity conditions can be well explained by cognitive load theory. According to this theory, individuals have limited cognitive resources, and appropriate external cues can reduce extraneous cognitive load and enhance processing efficiency (Sweller, 2011). This theoretical framework is highly consistent with the findings of this study: unfamiliar format imposes higher intrinsic cognitive load on readers, whereas inter-word spaces, as explicit segmentation cues, simplify processing units by clearly delineating word boundaries, reducing unnecessary cognitive consumption, and enabling cognitive resources to focus more effectively on core processes such as lexical recognition and semantic processing, ultimately leading to improved reading efficiency.

### 4.2. Verification and Analysis of Hypothesis H2

The findings of this study further support Hypothesis H2. A significant interaction exists between the word segmentation method and the format familiarity, specifically manifested as follows: When the format familiarity increases, the facilitating effect of inter-word spaces disappears. The findings directly support the theoretical expectation that there may be a trade-off between the format familiarity and the facilitative or interfering effects produced by the presence or absence of word boundary information—and these results are highly consistent with those from previous studies. Combined with the present training manipulation, we further infer that the 10-day systematic reversed reading training enabled participants to build stable internal abstract representations and develop a perceptual chunking mechanism for this atypical text format (D. H. Wang et al., 2023; D. H. Wang et al., 2024). In the untrained group with low format familiarity, readers heavily depended on external visual cues such as spaces to segment dense text information. After receiving continuous training, participants formed implicit expectations and structural representations of reversed text internally. Such internal representations greatly reduced readers’ dependence on external spacing segmentation cues, which in turn weakened and offset the facilitative effect of visual word boundaries. First, this result echoes the classic research perspective on how format familiarity and changes in visual cue dependency interact: as the familiarity of reading-related processing increases, reliance on external visual cues tends to decrease (Schotter et al., 2012). Second, the experimental results align with the central argument proposed by X. Li and Pollatsek (2020) that skilled readers rely more on internal linguistic representations than on external visual features, further substantiating the cross-script applicability of this view. At the same time, our findings provide new empirical support for the conclusion drawn by Gavril et al. (2021) that “during the development of reading skills, individuals’ reliance on low-level visual cues undergo systematic changes.” Moreover, this study adds cross-linguistic evidence to the finding by Chen et al. (2022) that “under conditions of low familiarity, explicit segmentation cues play a more prominent role” in Chinese reading. Specifically, the pattern observed in our study, where low familiarity relies heavily on inter-word spaces while high familiarity weakens this reliance, is consistent with the findings from Chinese reading research, further suggesting that this cognitive pattern may have universal applicability across different language systems.

From the perspective of processing mechanisms, the above-mentioned interaction and trade-off phenomena can be explained by the theory of automated reading processing. As Rayner (2009) points out, as readers accumulate reading experience or become more familiar with format, their lexical recognition processes gradually become automated. One of the key characteristics of automated processing is a significant reduction in reliance on explicit visual cues. This theoretical framework directly accounts for our study’ s findings: during the stage of low familiarity, readers’ processing of text format has not yet become automated, and lexical recognition and word segmentation still require substantial cognitive resources. Thus, readers actively rely on the external, explicit cue of inter-word spaces to reduce processing load and enhance efficiency. However, once familiarity increases and internal lexical recognition mechanisms become fully automated, external cues are no longer needed to facilitate efficient processing; consequently, the facilitating effect of inter-word spaces naturally diminishes and eventually disappears.

### 4.3. Supplementary Experimental Findings

It is worth noting that the finding in this study, that the increase in format familiarity completely negates the facilitating effect of spaces, differs somewhat from the observation made by Chen et al. (2022) in Chinese, where the facilitating effect was merely weakened. This difference can be attributed primarily to the inherent characteristics of the orthographic system. Specifically, Chinese, being a logographic writing system, features independent character forms with high semantic density; even at high levels of familiarity, spaces may still retain a subtle auxiliary role in confirming word boundaries. In contrast, Tibetan, as a phonetic script, relies heavily on explicit visual boundaries for lexical segmentation (D. H. Wang et al., 2024). For readers with lower familiarity, spaces serve as crucial cues for reducing cognitive load; however, once processing becomes automated through training, readers begin to perceive words as holistic perceptual chunks based on letter sequences. Under such circumstances, inserted spaces may instead disrupt this smooth, holistic perception or create conflicts with internal predictions (Perea & Acha, 2009). Meanwhile, the phonetic orthography exhibits a broader perceptual span; skilled readers can more efficiently leverage parafoveal information to extract multi-character units in a single glance (Y. Bai et al., 2008; Schotter et al., 2012). This makes the substitution effect of external cues even more pronounced in Tibetan. Consequently, in Tibetan reading, the internal automatic mechanisms completely override the influence of external visual cues, rather than merely partially attenuating it as in Chinese. This suggests that orthographic type plays an important moderating role in readers’ reliance on visual cues within the Sino-Tibetan language family. In addition, this study found that the format familiarity not only moderates the facilitative effect of inter-word spaces but also systematically influences the disruptive effect of anomalous spaces. Specifically, under unfamiliar format conditions, the interference caused by these anomalous spaces is relatively limited; whereas under familiar format conditions, their disruptive impact is significantly enhanced, as evidenced by slower reading speeds and longer fixation durations. This finding resonates with previous research on the processing costs associated with “predictive errors” in phonetic scripts and Chinese reading (Christofalos et al., 2024; Kuperberg & Jaeger, 2016; Perea & Acha, 2009). The underlying mechanism lies in the fact that as readers develop stable internal expectations about normal word structures, any visual information that violates these expectations triggers stronger processing conflicts (Haeuser & Kray, 2024; Pickering & Gambi, 2018), thereby increasing the real-time processing costs of reading. This indicates that the disruptive effects of both inter-word spaces and non-word spaces are also modulated by format familiarity in the current experimental context.

We further interpreted the magnitude of all significant effects via the equivalent correlation coefficient *r*. Most observed effects ranged from small to medium in size, which is a typical pattern in eye-tracking studies focusing on skilled adult reading (Rayner, 2009). Although no extremely large effects were detected, these results are still practically meaningful. The moderating relationship between format familiarity and word segmentation cues reveals a universal cognitive trade-off mechanism in script processing across writing systems (Christofalos et al., 2024).

### 4.4. Expanded Practical Applications of Research Findings

These results also yield valuable implications for the design of reading systems and reading instruction, especially for developing child readers. Drawing on relevant studies on Chinese reading (Chen et al., 2022; Y. Wang et al., 2022), we argue that flexible adaptation to varying visual formats is a key feature of mature reading ability. For young children in the early stages of reading acquisition, stable visual cues such as spacing serve as essential scaffolding to support text decoding. As reading proficiency develops, analogous to the improvement induced by our 10-day training, readers gradually shift their reliance from superficial visual formats to stable internal linguistic representations. In this sense, effective reading education should not only focus on training basic visual decoding skills, but also prioritize cultivating robust internal lexical representations that enable readers to adapt flexibly to diverse text presentations.

The present findings also yield meaningful practical implications for Tibetan reading instruction, literacy development and text layout design. For novice Tibetan readers, young learners and second-language learners who have not yet formed stable reading habits, inserting inter-word spaces as explicit word segmentation cues helps reduce reading-related cognitive load (L. Li et al., 2025). This layout design can support beginners with word segmentation and lexical processing, and promote early literacy development. For proficient Tibetan readers with high format familiarity, however, extra spaces will generate disruptive effects and impair reading efficiency, so the traditional unspaced format is more suitable. Accordingly, differentiated text presentation strategies are recommended: inter-word spaces can be adopted in teaching materials for novice and second-language learners, while traditional unspaced layout should be maintained for formal texts and reading materials targeting skilled readers. Such differentiated design fits the reading characteristics of different groups and optimizes overall reading performance.

### 4.5. Research Limitations

This study has several limitations. First, all participants were Tibetan university students. Given the differences in reading ability and cognitive characteristics between college students and the general population, the current findings cannot be directly generalized to broader reader groups. Second, there was an obvious gender imbalance in the sample, with far more female participants than males. Gender differences may exert potential influences on reading performance and eye movement patterns, which limits the representativeness of the results. Third, the reading intervention only lasted for 10 days. Although this short-term training successfully increased participants’ familiarity with the reversed text format, it cannot fully reflect stable long-term reading habits. Future research could recruit more diverse participants, balance the gender ratio, and adopt longer training sessions to further verify the present conclusions.

### 4.6. Future Research Directions

Based on the limitations summarized above, we put forward targeted directions for follow-up in-depth exploration. First, future research should expand participant recruitment scope to cover primary school students, middle school students, elderly Tibetan residents and Tibetan second-language learners of different age groups, balance the gender ratio of samples, and compare group differences in the trade-off effect between format familiarity and word segmentation cues to improve the external validity of relevant theories. Second, longer-term longitudinal training experiments can be designed to track the dynamic changes of eye-movement indicators over weeks or months of continuous reading practice, exploring the stable long-term evolution law of readers’ dependence on visual spacing cues. Third, multiple types of unfamiliar text formats can be set as experimental variables simultaneously, including reversed text, adjusted font specifications and altered line spacing, to verify whether the trade-off hypothesis can be stably reproduced under diverse unfamiliar visual presentation conditions. Fourth, follow-up research can further introduce individual difference variables such as reading fluency level, phonological awareness and orthographic sensitivity, to analyze how individual cognitive traits moderate the interactive relationship between format familiarity and inter-word spacing effects. Fifth, cross-Sino-Tibetan controlled experiments can be carried out with parallel Chinese and Tibetan experimental materials to systematically quantify how orthographic differences (logographic vs. phonetic) adjust the magnitude of spacing facilitation and interference effects under different familiarity levels, forming a more complete comparative framework for Sino-Tibetan reading cognition research. Furthermore, future studies could consider examining the processing pattern prior to training in the traditional writing/reading format, which would provide more convincing evidence regarding the training effects.

### 4.7. General Summary of Discussion

In summary, this study confirms that a dynamic trade-off exists between the facilitative effect of inter-word spaces and format familiarity during Tibetan reading. This finding tentatively extends the trade-off hypothesis derived from Chinese reading research to Tibetan, another language in the Sino-Tibetan family. The results reveal similar cognitive patterns of cue utilization between these two closely related languages, while also demonstrating unique processing features of Tibetan as a phonetic script. The present findings do not support broad generalization to all language systems, but provide an integrative perspective to understand word segmentation processing among Sino-Tibetan languages, and highlight the regulatory role of reading adaptability in using visual segmentation cues.

## 5. Conclusions

Under the conditions of this study, the following conclusions can be drawn: Under low format familiarity conditions, inter-word spaces significantly facilitate Tibetan reading; however, this facilitative effect disappears as format familiarity increases. In Tibetan reading, there is a trade-off between format familiarity and the facilitating effect of inter-word spaces.

## Figures and Tables

**Table 1:** Example Tibetan stimuli from the four conditions used in the experiment.

Word Segmentation	Sentence Example
Unspaced	རྒྱས།ཡོན་ཤེས་པས་བཀླགས་པ་གསར་དེབ་ཀྱི་བོད་མས་སློབ
Inter-word spaced	རྒྱས ཡོན་ཤེས པས་བཀླགས པ་གསར དེབ ཀྱི་བོད མས་སློབ
Inter-character spaced	རྒྱས ཡོན ཤེས པས བཀླགས པ གསར དེབ ཀྱི བོད མས སློབ
Non-word spaced	རྒྱས།ཡོན ཤེས་པས བཀླགས པ གསར་དེབ་ཀྱི བོད་མས སློབ

Note: The above sentence means “The student has expanded their knowledge by reading the new Tibetan book.”.

**Table 2:** Means and standard deviations of eye movement measures across conditions.

Eye Movement Measures	Unfamiliar Format				Familiar Format			
	Unspaced	Inter-Word Spaced	Inter-Character Spaced	Non-Word Spaced	Unspaced	Inter-Word Spaced	Inter-Character Spaced	Non-Word Spaced
RR	4.56 (1.77)	4.65 (1.68)	4.64 (1.66)	4.11 (1.45)	5.15 (1.65)	5.07 (1.63)	5.21 (1.66)	4.96 (1.58)
AFD	256.31 (32.76)	240.45 (34.21)	246.18 (33.09)	251.31 (34.18)	142.72 (66.59)	145.64 (77.34)	146.49 (87.65)	150.61 (82.43)
TRT	6829 (2904)	6581 (2659)	6612 (2754)	7430 (3080)	5777 (1951)	5869 (2059)	5750 (2023)	5966 (2753)
FSP	70.43% (0.16)	72.91% (0.13)	69.90% (0.16)	71.49% (0.16)	77.83% (0.11)	77.76% (0.10)	77.19% (0.11)	78.45% (0.11)
RSC	6.61 (4.50)	6.13 (4.16)	6.32 (4.31)	6.68 (4.35)	4.51 (3.26)	4.71 (3.39)	4.57 (3.46)	4.80 (3.35)
TNF	22.35 (8.77)	22.19 (8.53)	22.11 (8.30)	22.35 (8.53)	19.67 (6.62)	20.25 (6.79)	20.06 (6.62)	20.21 (6.68)

Note: The data in the table are presented as *M* (*SD*), where RR stands for reading speed (characters/s), AFD for average fixation duration (ms), TRT for total sentence reading time (ms), FSP for forward saccade probability (%), RSC for number of regressions (times), and TNF for total number of fixations (times). The same applies below.

**Table 3:** Fixed effect estimates for each eye movement measure.

Eye Movement Measures		Intercept	Format-Familiarity	Word Segmentation 1	Word Segmentation 2	Word Segmentation 3	Interaction 1	Interaction 2	Interaction 3	Comparison 1	Comparison 2	Comparison 3	Comparison 4	Comparison 5	Comparison 6
RR	*b*	1.45	0.14	0.03	0.02	−0.1	−0.05	−0.01	0.06	−0.03	−0.02	0.10	0.02	−0.01	0.04
	*SE*	0.04	0.05	0.01	0.01	0.01	0.02	0.02	0.02	0.01	0.01	0.01	0.01	0.01	0.01
	*t*	38.67 ***	2.67 **	2.30 *	1.76	−7.81 ***	−2.38 *	−0.56	3.38 ***	−2.31	−1.76	7.81 ***	1.11	−0.84	2.55
AFD	*b*	5.54	−0.69	−0.07	−0.04	−0.02	0.08	0.05	0.08	0.07	0.04	0.02	−0.02	−0.01	−0.05
	*SE*	0.03	0.04	0.02	0.02	0.02	0.02	0.02	0.02	0.01	0.01	0.01	0.02	0.02	0.02
	*t*	205.93 ***	−17.00 ***	−4.43 ***	−2.80 **	−1.48	3.45 ***	1.94	3.18 **	4.43 ***	2.80 *	1.47	−0.84	1.00	−2.90 *
TRT	*b*	8.75	−0.14	−0.03	−0.02	0.09	0.04	0.01	−0.05	0.03	0.02	−0.09	−0.01	0.01	−0.03
	*SE*	0.04	0.05	0.01	0.01	0.01	0.02	0.02	0.02	0.01	0.01	0.01	0.01	0.01	0.01
	*t*	224.26 ***	−2.58 *	−2.22 *	−1.96	6.84 ***	2.12 *	0.75	−2.79 **	2.22	1.96	−6.84 ***	−0.84	0.77	−2.47
FSP	*b*	0.95	0.42	0.13	−0.02	0.07	−0.13	−0.03	−0.03	1.13	1.05	0.98	0.99	1.03	0.96
	*SE*	0.08	0.12	0.03	0.03	0.03	0.04	0.04	0.04	0.03	0.02	0.02	0.03	0.03	0.03
	*t*	11.57 ***	3.52 ***	4.50 ***	−0.77	2.42 *	−3.15 **	−0.65	−0.69	5.05 ***	2.13	−0.91	−0.44	0.84	−1.31
RSC	*b*	1.85	−0.32	−0.05	−0.04	0.02	0.08	0.03	0.03	0.95	0.98	1.00	0.96	0.96	0.98
	*SE*	0.06	0.09	0.02	0.02	0.02	0.03	0.03	0.03	0.01	0.01	0.01	0.01	0.01	0.01
	*t*	29.60 ***	−3.54 ***	−2.11 *	−1.63	1.06	2.39 *	0.90	0.84	−3.70 **	−0.27	−1.24	−2.52	−1.60	−2.56
TNF	*b*	3.09	−0.10	0.00	0.01	−0.01	0.03	0.02	0.02	0.01	0.01	−0.01	−0.03	−0.02	−0.03
	*SE*	0.04	0.05	0.01	0.01	0.01	0.02	0.02	0.02	0.01	0.01	0.01	0.01	0.01	0.01
	*t*	86.20 ***	−2.07 *	−0.04	−0.22	0.26	1.64	1.15	1.32	0.04	0.22	−0.25	−2.18	−1.35	−2.02

Note: *** *p* < 0.001, ** *p* < 0.01, * *p* < 0.05.

## Data Availability

The original contributions presented in the study are available from the corresponding authors upon reasonable request.
